# Struggling with Self-Doubt: Impostor Phenomenon and Mental Health among Medical Students at Universitas Sumatera Utara Medan, Indonesia

**DOI:** 10.2174/0117450179397570250706100944

**Published:** 2025-07-11

**Authors:** Ruhut Sion Simanjuntak, Rina Amelia, Elmeida Effendy, Yuki Yunanda

**Affiliations:** 1 Faculty of Medicine, Universitas Sumatera Utara, Medan, Indonesia; 2 Department of Community Medicine, Faculty of Medicine, Universitas Sumatera Utara, Medan, Indonesia; 3 Department of Psychiatry, Faculty of Medicine, Universitas Sumatera Utara, Medan, Indonesia

**Keywords:** Impostor phenomenon, Depression, Anxiety, Low self-esteem, Medical student, Depression

## Abstract

**Introduction:**

Impostor Phenomenon (IP) is a common condition experienced by medical students and professionals who face high academic pressures and competitive environments, and is thought to be associated with other psychological conditions, such as anxiety, depression, and low self-esteem. Factors, such as gender and academic year, are also suspected to influence the development of IP experienced by individuals. This study aimed to analyze factors associated with IP among students at the Faculty of Medicine, Universitas Sumatera Utara.

**Methods:**

This analytical study employed a cross-sectional design. Variables assessed included gender, academic year, depression, anxiety, and self-esteem. Several questionnaires were used for data collection, such as CIPS, PHQ-9, GAD-7, and RSES questionnaires. Data analysis utilized Chi-square tests to investigate the relationship between each independent variable and Poisson regression tests to evaluate the correlation between all independent variables using the Statistical Package for the Social Sciences (SPSS Inc.).

**Results:**

A total of 347 students participated, revealing that 58.8% of medical students experienced IP, with a greater prevalence among females and the highest prevalence among second-year students. Data analysis revealed a significant association between IP and symptoms of depression (PR = 1.530; 95% CI = 1.229-1.904; P < 0.001), anxiety (PR = 1.317; 95% CI = 1.095-1.583, P = 0.003), and low self-esteem (PR = 1.237; 95% CI = 1.066-1.449; P = 0.008).

**Discussion:**

This study reinforces that psychological factors, including depression, anxiety, and low self-esteem, are strongly associated with the impostor phenomenon (IP) among medical students, while gender and academic year are not. The findings highlight the need for early mental health support to help students manage self-doubt and academic stress.

**Conclusion:**

The study highlights a high prevalence of IP among medical students, with a significant relationship between IP, depression, anxiety, and self-esteem.

## 1 INTRODUCTION

The Impostor Phenomenon (IP), also referred to as impostor syndrome or impostorism, is a psychological pattern in which individuals fail to internalize their accomplishments, question their own abilities, and attribute them to external influences, such as luck, despite having objective evidence supporting their abilities or accomplishments [1].

This phenomenon was first introduced by Clance and Imes in 1978 to describe self-doubt behavior in high-achieving women [1]. Those experiencing IP often harbor a persistent fear of being unmasked as frauds. They often reject positive feedback, strive for unattainable perfection, and set unrealistically high standards [1, 2]. Low self-esteem can further amplify these struggles and can lead to high psychological distress, including anxiety, depression, burnout, and impaired cognitive and behavioral performance [3, 4].

While initial studies focused on high-achieving women, recent findings reveal that every person is prone to experiencing the impostor syndrome, including individuals in the medical field, such as doctors, physicians, surgeons, and medical students. The prevalence of IP varies widely depending on the screening tools and cutoff points used in the study procedure [5]. The prevalence among medical students ranges from 25.5% to 62.8% [4, 6-10].

Several factors can contribute to someone developing impostor feelings. One of those is the transition to a new environment. Past research found that first-year students often experience heightened IP due to role transitions and the academic pressure associated with adapting to a new environment [11, 12]. Transitions are also a part of the majority of medical school systems, in which students transition to the clinical phase after completing their preclinical phase, which can bring a completely new environment and the need for adaptation. Studies have found a high incidence of IP experiences among students during this period [13, 14]. Medical school can also be too competitive; from the beginning of the medical journey to the admission process, medical schools accept top performers in high school. These top performers may have already developed the impostor syndrome feeling even before being accepted. While they find themselves surrounded by other top performers, they can doubt themselves even more and pressure themselves to perform better [2]. Other factors, such as gender and age, have also been documented as contributing to high IP experience [5], although there are varying study results regarding these factors. While it was first introduced in women, current studies have shown no difference in rates between men and women, especially in professional areas [6, 15-19]. Meanwhile, age is considered to be negatively correlated with IP, with an increase in age associated with lower impostor feelings [12, 19].

In terms of impact, IP mainly affects the psychological well-being of an individual. Depression and anxiety are the most documented psychological distress related to IP [5]. Previous studies also found its association with burnout, cynicism, depersonalization, emotional exhaustion, low self-esteem, neuroticism, somatic symptoms, social dysfunctions, quitting medical school, and suicidal ideation [4-6, 13].

Recent large-scale research has provided additional insight into the broader mental health context relevant to our findings. Song *et al.* (2023), in their nationwide study among over 2.8 million Koreans from 2009 to 2021, reported a concerning increase in sadness and a decline in average sleep duration, particularly during the COVID-19 pandemic. Interestingly, despite these trends, the use of counseling services did not increase proportionally, highlighting a persistent treatment gap. This underscores the potential for unaddressed depressive symptoms among populations under chronic stress, such as medical students [20]. Complementing this, Kandola *et al.* (2023) conducted an umbrella review consolidating evidence that physical activity substantially reduces the risk of mental health complications, including depression and anxiety. This reinforces the idea that promoting regular physical activity could serve as a non-pharmacological, accessible intervention for reducing psychological distress and potentially mitigating symptoms of the impostor phenomenon in high-stress academic environments [21].

This study aimed to assess the prevalence of impostor phenomenon (IP) among medical students at Universitas Sumatera Utara, given its high prevalence and detrimental effects on individual well-being. It sought to compare prevalence by gender and academic year, as well as to analyze the correlation between IP and mental health issues, thereby highlighting the significance of IP within Indonesian culture, in light of the limited research on this phenomenon among medical students in Indonesia.

## 2 MATERIALS AND METHODS

### 2.1 Design And Participants

The study adopted a quantitative analytical cross-sectional design to analyze the relationship between students’ characteristics, which include gender, academic year, level of depression, anxiety, and self-esteem, as the independent variables, and IP as the dependent variable. Data were collected through an online questionnaire survey conducted among medical students at the Faculty of Medicine, Universitas Sumatera Utara, including both undergraduate students and students in clerkship training. The data collection period took place from August to October 2024, with eligibility criteria ensuring all respondents were active students within these categories. Participation in this study was voluntary; participants were not obligated to participate. Earlier, the researcher provided an explanation regarding the ongoing research and their active participation in it. Subsequently, patients who gave their consent would sign the informed consent form.

To determine the minimum required sample size, the Slovin formula was applied with a 5% margin of error, and the minimum sample size estimated was 316. Invitations were sent to over 1500 students, of which 363 responded. After excluding the unreliable responses, identified by checking the exclusion criteria, which included a self-reported history of psychological illnesses that could confound the relationship between the variables being studied, a total of 347 samples were included in the final analysis.

### 2.2 Instrument

The questionnaire used as the instrument in this study was the 20-item Clance Impostor Phenomenon Scale (CIPS). The questionnaire had a 5-point Likert scale (Always: 5, usually: 4, sometimes: 3, rarely: 2, never: 1). A score of 62 or higher was used to indicate the presence of IP. This threshold was determined based on a study by Holmes, which found a minimum false positive and a zero false negative using 62 as the cutoff point [22]. The CIPS questionnaire demonstrated excellent internal consistency, with a Cronbach’s alpha coefficient of 0.96 [23].

Depression was measured using the Indonesian version of the Patient's Health Questionnaire-9 (PHQ-9) [24], which also demonstrated strong internal consistency with a Cronbach's alpha of 0.885. Anxiety was measured with the Indonesian version of the Generalized Anxiety Disorder-7 (GAD-7) questionnaire, which also had a strong internal consistency, shown by Cronbach's alpha coefficient of 0.87 [25]. PHQ-9 and GAD-7 are both 4-point Likert scale questionnaires. They were used to measure depression and anxiety symptoms experienced by individuals in the past two weeks before filling out the questionnaire. Both also have the same cutoff point, a score of 10 or higher, to indicate moderate to severe depression or anxiety [26, 27]. Then, self-esteem was assessed using the 10-item Rosenberg Self-Esteem Scale (RSES) as a 4-point Likert scale questionnaire. Cronbach's alpha coefficient of this questionnaire ranges from 0.77 to 0.88. A score lower than 15 indicates an individual with low self-esteem [28, 29].

This study adhered to the SAGER (Sex and Gender Equity in Research) guidelines to ensure proper reporting of sex and gender-based analyses. Gender was considered a key variable in both data collection and analysis. Results were disaggregated by gender, and statistical comparisons were conducted to examine potential differences between the groups. The completed SAGER Checklist is provided as a supplementary file for transparency and compliance [30].

### 2.3 Statistical Analysis

We conducted univariate analysis to determine the prevalence and demographic characteristics. Bivariate analysis was carried out to examine the relationship between each independent variable, such as gender, academic year, depression, anxiety, and self-esteem, and the dependent variable, IP, using the Chi-Square tests (p < 0.05). The multivariate analysis, which employed Poisson Regression Tests, was also performed to evaluate the correlation between all independent variables that had a significant relationship with the dependent variable, as determined by the chi-square test (p < 0.05). Statistical analyses were performed using the Statistical Package for the Social Sciences (SPSS Inc.), Version 29.0.

## 3 RESULTS

The participant demographics are depicted in Table **1** Out of 347 participants in this study, the majority were females (64.6%) and fourth-year medical students (22.8%). The prevalence of IP in this study was 58.8%. The majority of the participants were not experiencing depression (28.8%) or anxiety (37.5%), and had good self-esteem (80.1%), as depicted in Table **2**.

The prevalence of the impostor phenomenon and associated mental health issues is depicted in Table **3** Regarding mental health issues, we found that females had slightly higher rates of depression. Meanwhile, men had higher rates of anxiety and lower self-esteem. We also found second-year students with the highest rate of mental health problems, with an IP rate of 67.9%, a depression rate of 58.5%, an anxiety rate of 37.7%, and a low self-esteem rate of 24.5%.

Table **4** shows that IP was higher in females and second-year students. No significant relationship was found between gender and years of study, nor was the IP experienced by medical students. However, a relationship was found between depression, anxiety, and low self-esteem with IP. A positive correlation was found between depression and anxiety with IP; students with high depression and anxiety scores tended to have higher IP scores. Meanwhile, the correlation between self-esteem and IP was negative, in which students with low self-esteem tended to have a high impostor feeling.

Table **5** shows the results of the multivariate analysis between significant factors associated with the impostor phenomenon, as indicated by the chi-square test with a p-value lower than 0.05. The result shows that depression has the greatest prevalence ratio, followed by anxiety and low self-esteem.

## 4 DISCUSSION

IP was initially introduced in studies on high-achieving women to explain their difficulty in internalizing success. Although initially associated with women, IP is linked to gender stigma awareness, with higher awareness making individuals, especially women, more vulnerable to IP [31-33]. Our study found that 59.8% of women and 56.9% of men reported experiencing IP. While women exhibited slightly higher rates, this difference was not statistically significant. These findings align with prior research [6, 8], which also concluded that IPs are not gender-dependent. This suggests that both men and women are equally susceptible to IP, indicating a shift away from traditional assumptions that primarily associate IP with women. Our findings also indicate broader societal progress in reducing gender bias. The diminishing link between IP and gender reflects trends toward greater gender equality, challenging stereotypes about women’s abilities. These shifts are evident in the growing representation of women in traditionally male-dominated fields, such as medicine and leadership roles within academic departments. Such progress fosters environments where both men and women experience similar psychological challenges, such as IP.

Regarding the academic year, our study aligns with prior research showing no significant link between the academic year and IP prevalence [7]. However, preclinical students (first-year to fourth-year students) reported higher rates of IP compared to clinical students (fifth-year and sixth-year students), with second-year students exhibiting the highest rates. This could be attributed to their limited medical knowledge and skills, combined with the demands of adaptation to independent learning methods typical in medical education. Struggles in adjusting during the first year may carry over, contributing to higher IP rates in second-year students. Additionally, second-year students reported the highest rates of depression (58.5%), anxiety (37.7%), and low self-esteem (24.5%). These may be partly influenced by their increased involvement in extracurricular activities, which become more active in the second year. Nair *et al.* also found that mental health challenges among medical students are not static and tend to intensify during key transition periods in medical education. Specifically, the shift from preclinical to clinical training, often occurring in the second year, is associated with increased stress, anxiety, and feelings of inadequacy. Factors contributing to this include heightened academic demands, exposure to clinical environments, and a sense of loss of control, all of which can exacerbate psychological distress during this critical phase of medical training [34]. This finding draws attention to the compounding impact of extracurricular activities, which, while beneficial for personal growth and professional skills, can inadvertently contribute to stress and reduced mental well-being when they overwhelm students’ capacity to manage their time and energy.

A significant relationship was observed between depression, anxiety, and IP. Among students with moderate to severe depression, 84% experienced IP, while 84.6% of those with moderate to severe anxiety also reported IP. High societal expectations of medical students, particularly those from prestigious institutions, combined with competition for further education and employment opportunities, place immense pressure on medical students to excel. The constant need to achieve excellent results in frequent exams and assessments can intensify this pressure and lead to depression and anxiety among students. It can trigger or exacerbate IP, especially if students doubt their ability to meet targets or expectations. The findings of our study align with prior research [15, 16, 35-37], which identified positive correlations between IP and both depression and anxiety. The causal relationship between IP and depression or anxiety remains unclear and may be bidirectional or form a cyclical interaction. Self-doubt, high standards, and the constant need to prove competence in individuals with IP can lead to depression and anxiety, while experiencing depression or anxiety can increase vulnerability to or worsen IP. However, as this study is cross-sectional, it cannot establish causation between IP, depression, and anxiety. Future longitudinal research could provide greater clarity on these relationships and help determine causality. Nonetheless, the observed correlations underscore the need for mental health support in academic environments.

Cultural factors also play a significant role in shaping the experience of IP among Indonesian medical students. In Indonesian society, collectivist values emphasize group harmony, familial expectations, and social conformity. These cultural norms can intensify feelings of inadequacy and self-doubt in students who perceive themselves as not meeting the high standards set by their families and communities. For instance, authoritarian parenting styles, characterized by high expectations and low emotional support, have been linked to higher instances of IP among Indonesian first-year students, suggesting that early familial influences can contribute to the development of IP [38].

Low self-esteem was reported by 19.9% of respondents, consistent with findings among Saudi medical students (17.6%) [39]. Among those with low self-esteem, 84.1% experienced IP. Our analysis identified low self-esteem as a significant factor associated with IP, consistent with prior studies [9, 40], which highlighted a negative correlation between IP and self-esteem. Low self-esteem could be linked to IP’s “discount” dimension. It is a domain that describes individuals with IP who tend to downplay their achievements and doubt their abilities [41]. In the context of IP, low self-esteem frequently coexists with maladaptive perfectionism, characterized by excessive self-criticism and an inability to meet perceived standards, thereby reinforcing impostor feelings [42].

Additionally, this study did not find a significant relationship between gender or academic year and depression, anxiety, or low self-esteem. However, low self-esteem was found to be significantly associated with increased symptoms of depression and anxiety, reinforcing the interconnectedness of these variables. These findings call for targeted interventions to address low self-esteem, as improving self-perception may help reduce the impact of depression, anxiety, and IP.

Recent studies have emphasized the interconnectedness between physical health indicators and psychological distress, adding valuable context to our findings on depression and anxiety. Kim *et al.* (2023), in their analysis of multicontinental databases, reported that reduced grip strength is potentially indicative of depression, highlighting a tangible, physiological marker that could aid in early identification of at-risk individuals. This is particularly relevant in academic environments where psychological symptoms may be overlooked or underreported [43]. Additionally, Lee *et al.* (2023) examined national trends in sadness, suicidality, and pandemic-related mental health risk factors among South Korean adolescents from 2005 to 2021. Their findings revealed a long-term increase in emotional distress, exacerbated during the COVID-19 pandemic, which aligns with the elevated levels of depression and anxiety observed in our medical student population [44]. These studies collectively underscore the importance of integrating physical and mental health screening to more comprehensively address the psychological burden associated with IP.

To mitigate the effects of IP, institutions should foster supportive environments that normalize discussions about self-doubt and mental health. Mentorship or workshop programs can help students navigate challenges. Additionally, balancing academic demands with extracurricular opportunities and ensuring access to mental health resources can help students manage psychological distress effectively. Incorporating cultural sensitivity into these interventions and promoting a more collaborative rather than competitive learning environment could help mitigate the effects of IP. Addressing these factors may enhance students’ well-being and reduce the prevalence of IP, creating a healthier academic experience.

However, the study has limitations, including the reliance on self-reported data, which can introduce social desirability bias. Medical students, in particular, may underreport symptoms of depression or anxiety due to stigma or the fear of being perceived as weak or incapable. To mitigate this, we ensured participant anonymity and emphasized confidentiality throughout the study. Nonetheless, we recommend that future studies employ a mixed-methods approach by incorporating objective assessments, interviews, or observational data to validate and enrich self-reported findings. Additionally, the cross-sectional design prevents establishing causality between IP and psychological factors. Future research should explore longitudinal studies and larger sample sizes to enhance generalizability and better understand the long-term effects of IP on students' well-being.

## CONCLUSION

The study highlights a high prevalence of the Impostor Phenomenon (IP) among medical students, with a significant relationship between IP, depression, anxiety, and self-esteem. Findings indicate that students with higher levels of depression and anxiety tend to experience stronger impostor feelings, whereas those with lower self-esteem exhibit higher IP scores. The results emphasize the need for mental health interventions tailored to students experiencing these psychological challenges.

## Figures and Tables

**Table 1:** Demographics of participants.

**Variables**	**Categories**	**N (%)**
Gender	MaleFemale	123 (35.4%)224 (64.6%)
Academic Year	1^st^ year student2^nd^-year student3^rd^-year student4^th^-year student5^th^-year student6^th^-year student	61 (17.6%)53 (15.3%)51 (14.7%)79 (22.8%)51 (14.7%)52 (15%)

* N = percentage.

**Table 2:** Prevalence of impostor phenomenon and associated mental health issues.

**Variables**	**Categories**	**N (%)**
Impostor Phenomenon	YesNo	204 (58.8%)143 (42.2%)
Depression	NoMildModerateModerately severeSevere	100 (28.8%)99 (28.5%)92 (26.5%)41 (11.8%)15 (4.3%)
Anxiety	NoMildModerateSevere	130 (37.5%)113 (32.6%)72 (20.7%)32 (9.2%)
Self esteem	GoodLow	278 (80.1%)69 (19.9%)

* N = percentage.

**Table 3:** Prevalence of impostor phenomenon and associated mental health issues.

**Factors**	**Depression**		**Anxiety**		**Self-esteem**	
**Yes (%)**	**No (%)**	**Yes (%)**	**No (%)**	**Low (%)**	**Good (%)**
Gender						
Male	51 (41.5%)	72 (58.5%)	41 (33.3%)	82 (66.7%)	31 (25.2%)	92 (74.8%)
Female	97 (43.3%)	127 (56.7%)	63 (28.1%)	161 (71.9%)	38 (17.0%)	186 (83.0%)
Academic year						
1^st^-year student	29 (47.5%)	32 (52.5%)	22 (36.1%)	39 (63.9%)	14 (23.0%)	47 (77.0%)
2^nd^-year student	31 (58.5%)	22 (41.5%)	20 (37.7%)	33 (62.3%)	13 (24.5%)	40 (75.5%)
3^rd^-year student	18 (35.3%)	33 (64.7%)	14 (27.5%)	37 (72.5%)	7 (13.7%)	44 (86.3%)
4^th^-year student	38 (48.1%)	41 (51.9%)	24 (30.4%)	55 (69.6%)	12 (15.2%)	67 (84.8%)
5^th^-year student	15 (29.4%)	36 (70.6%)	13 (25.5%)	38 (74.5%)	12 (23.5%)	39 (76.5%)
6^th^-year student	17 (32.7%)	35 (67.3%)	11 (21.2%)	41 (78.8%)	11 (21.2%)	41 (78.8%)

**Table 4:** Factors associated with the impostor phenomenon.

**Factors**	**Impostor (n = 204)**		**Non-Impostor (n =143)**		**Prevalence Ratio** **(95% Confidence Interval)**	**p-Value**
**N**	**%**	**N**	**%**
Gender						
Male	70	56.9	53	43.1	1.051 (0.871-1.268)	0.598
Female	134	59.8	90	40.2		
Academic year						
1^st^-year student	36	59.0	25	41.0	1.005 (0.798-1.265)	0.484
2^nd^-year student	36	67.9	17	32.1	1.189 (0.964-1.466)	
3^rd^-year student	31	60.8	20	39.2	1.040 (0.818-1.323)	
4^th^-year student	47	59.5	32	40.5	1.016 (0.825-1.250)	
5^th^-year student	29	56.9	22	43.1	0.962 (0.744-1.244)	
6^th^-year student	25	48.1	27	51.9	0.792 (0.589-1.066)	
Depression						
Yes	119	80.4	29	19.6	1,882 (1.573-2.252)	
No	85	42.7	114	57.3		<0.0001
Anxiety						
Yes	88	84,6	16	15,4	1.772 (1.518-2.069)	<0.0001
No	116	47.7	127	52.3		
Self-esteem						
Low	58	84.1	11	15.9	1,601 (1,375-1,863)	<0,001
Good	146	52,5	132	47.5		

**Table 5:** Poisson regression of factors associated with the impostor phenomenon.

**Variables**	**Prevalence Ratio**	**95% C.I.**		**p-Value**
**Lower**	**Upper**
Depression	1.530	1.229	1.904	<0.001
Anxiety	1.317	1.095	1.583	0.003
Low self-esteem	1.237	1.066	1.449	0.008

## Data Availability

The data supporting the findings of the article is available in the FigShare at [https://figshare.com/], [DOI: 10.6084/m9.figshare.29551112].

## LIST OF ABBREVIATIONS

CIPS: = Clance Impostor Phenomenon Scale
COVID-19: = Coronavirus Disease 2019
GAD-7: = Generalized Anxiety Disorder-7
GOS: = Glasgow Outcome Scale
GAD: = Generalized Anxiety Disorder
ICP: = Intracranial Pressure
IP: = Impostor Phenomenon
PHQ-9: = Patient Health Questionnaire-9
RSES: = Rosenberg Self-Esteem Scale
SPSS: = Statistical Package for the Social Sciences
USU: = Universitas Sumatera Utara

## STANDARDS OF REPORTING

STROBE guidelines were followed.
